# Worms take to the slo lane: a perspective on the mode of action of emodepside

**DOI:** 10.1007/s10158-012-0133-x

**Published:** 2012-04-27

**Authors:** Lindy Holden-Dye, Anna Crisford, Claudia Welz, Georg von Samson-Himmelstjerna, Robert J. Walker, Vincent O’Connor

**Affiliations:** 1 Centre for Biosciences, University of Southampton, Building 85, University Road, Southampton, SO17 1BJ UK; 2 Bayer HealthCare Animal Health, Bayer Animal Health GmbH, GDD-AH-PARA-AR, 6700 Monheim, Germany; 3 Institute for Parasitology and Tropical Veterinary Medicine, Freie Universität Berlin, Königsweg 67, 14163 Berlin, Germany

**Keywords:** Cyclo-octadepsipeptide, *C. elegans*, *Ascaris suum*, Anthelmintic, BK channel

## Abstract

The cyclo-octapdepsipeptide anthelmintic emodepside exerts a profound paralysis on parasitic and free-living nematodes. The neuromuscular junction is a significant determinant of this effect. Pharmacological and electrophysiological analyses in the parasitic nematode *Ascaris suum* have resolved that emodepside elicits a hyperpolarisation of body wall muscle, which is dependent on extracellular calcium and the efflux of potassium ions. The molecular basis for emodepside’s action has been investigated in forward genetic screens in the free-living nematode *Caenorhabditis elegans*. Two screens for emodepside resistance, totalling 20,000 genomes, identified several mutants of *slo*-*1*, which encodes a calcium-activated potassium channel homologous to mammalian BK channels. *Slo*-*1* null mutants are more than 1000-fold less sensitive to emodepside than wild-type *C. elegans* and tissue-specific expression studies show emodepside acts on SLO-1 in neurons regulating feeding and motility as well as acting on SLO-1 in body wall muscle. These genetic data, combined with physiological measurements in *C. elegans* and the earlier physiological analyses on *A. suum*, define a pivotal role for SLO-1 in the mode of action of emodepside. Additional signalling pathways have emerged as determinants of emodepside’s mode of action through biochemical and hypothesis-driven approaches. Mutant analyses of these pathways suggest a modulatory role for each of them in emodepside’s mode of action; however, they impart much more modest changes in the sensitivity to emodepside than mutations in *slo*-*1.* Taken together these studies identify SLO-1 as the major determinant of emodepside’s anthelmintic activity. Structural information on the BK channels has advanced significantly in the last 2 years. Therefore, we rationalise this possibility by suggesting a model that speculates on the nature of the emodepside pharmacophore within the calcium-activated potassium channels.

## Receptors and effectors for emodepside

Emodepside is a semi-synthetic derivative of the cyclo-octadepsipeptide PF1022A, a secondary metabolite of the fungus *Rosellinia* spp. PF1022. It is effective against a wide-range of parasitic nematodes and has anthelmintic-resistance breaking properties suggesting a novel mode of action, for review see (Krucken et al. 2012). A key aspect of its efficacy is its ability to interfere with neuromuscular transmission in parasitic nematodes, an effect that was shown to be both calcium- and potassium dependent (Willson et al. 2003). It also inhibits neuromuscular function in the free-living nematode *C. elegans*, disrupting feeding, egg-laying and motility (Bull et al. 2007). The inhibition of motility leads to complete paralysis and is consistent with dysfunction at the neuromuscular junction. Observation of the flaccid paralysis triggered by emodepside suggests that it inhibits muscle contraction. This could arise through a direct effect on muscle and/or an indirect effect through the excitatory neural network that drives muscle contraction. Regardless of the locus of the effector for paralysis in *C. elegans*, this behavioural effect provides a very tractable assay for forward and reverse genetics and thus it has been used to identify the molecular determinants of emodepside’s anthelmintic action.

The ability of emodepside to break the resistance of parasitic nematodes to other anthelmintic agents (von Samson-Himmelstjerna et al. 2005) had already indicated that it exerts its actions through a novel target(s). This important prediction was confirmed when it was shown that SLO-1, a calcium-activated potassium channel, is required for the effect of emodepside in *C. elegans* (Guest et al. 2007), and this observation was later confirmed for parasitic nematodes (Welz et al. 2011). SLO-1 potassium channels belong to a family of high-conductance ion channels, designated BK for ‘big K^+^ conductance’, that are activated by changes in membrane voltage, that is, depolarisation and by increased cytoplasmic calcium ion concentration. This dual regulation confers on them a key role in neuronal, muscle and endocrine function throughout the animal phyla (Salkoff et al. 2006). In *C. elegans*, *slo*-*1* is expressed in both the nervous system and body wall muscle and the *slo*-*1* null mutant has pharyngeal, motility and egg-laying phenotypes, indicating it has a role in regulating these behaviours (Bull et al. 2007; Holden-Dye et al. 2007). This provides a locus for the disruption of these behaviours by emodepside. Experiments in which wild-type *slo*-*1* was re-introduced into the *slo*-*1* null mutant *C. elegans* in a tissue-specific manner have provided evidence that the emodepside-induced paralysis requires a contribution from neuronal and body wall muscle to exert full inhibition of locomotion (Guest et al. 2007). This same experimental approach was used to introduce the mammalian homologue of *slo*-*1, kcnma1*, into the *slo*-*1* null *C. elegans* mutant. Expression of the human channel rescued *slo*-*1* deficient behaviours in a similar fashion to expression of the native *C. elegans* channel *slo*-*1* (Crisford et al. 2011), thus suggesting that it is possible to functionally reconstitute the mammalian channel in *C. elegans*. However, when worms expressing either *slo*-*1* or *kcnma1* were compared with respect to the efficacy of emodepside to inhibit motility or pharyngeal activity, those expressing the nematode channel were at least 10-fold more sensitive to emodepside than worms expressing the human channel. This observation has been reinforced by investigations in which the same approach was used to compare the effect of emodepside on SLO-1 channels from several parasitic nematodes (Welz et al. 2011). These experiments confirmed the sensitivity of the parasitic nematode *slo*-*1* to emodepside. This suggests that structural or conformational differences between the calcium-activated potassium channel isoforms may contribute to emodepside’s selective action on the parasite (Crisford et al. 2011). Comparisons of its efficacy at different isoforms of the calcium-activated potassium channel might provide important clues to its molecular target within the calcium-activated potassium channels. Recently, it has been reported that there are at least twelve splice variants of *C. elegans slo*-*1* (Glauser et al. 2011) and that the presence of different exons impacts on the activation of the channel and its sensitivity to intracellular calcium ions (Johnson et al. 2011). Thus, another strand to consider is the possibility that differences in alternative splicing of *slo*-*1* between species might influence emodepside sensitivity.

An important feature of the in vivo pharmacological experiments described above is that they provide a direct quantitative comparison of the susceptibility of wild-type, mutant and transgenic *C. elegans* to emodepside. The results from these studies can further inform on putative receptors and pathways that might contribute to emodepside’s effect. Predominant among these candidates is the phylogenetically conserved latrophilin receptor. Latrophilin was identified as a candidate receptor for cyclo-octadepsipeptides by expression cloning from *Haemonchus contortus* (Saeger et al. 2001). It is a Class B G-protein-coupled receptor, so-called, because it binds the black widow spider toxin, latrotoxin (Lelianova et al. 1997). The mammalian version of this receptor is implicated in regulating synaptic function (Silva et al. 2011). Latrophilins have been identified in parasitic nematodes and these present another interesting target with respect to emodepside (Krüger et al. 2009). The significance of latrophilin in the mode of action of emodepside has been investigated by gene knockdown through RNAi, and gene knockouts in *C. elegans* (Willson et al. 2004). In a latrophilin null mutant (carrying deletions in both latrophilin genes, *lat*-*1* and *lat*-*2*), there is a 20-fold reduction in the sensitivity of the pharyngeal system to emodepside. The role for latrophilin appears reinforced by the comparative analysis of emodepside sensitivity in worms that are genetically deficient in key downstream pathways that might be activated by latrophilin receptors (Willson et al. 2004; Amliwala 2005; Bull 2007). Mutations in these genes confer up to a 50-fold reduction in sensitivity to the effects of emodepside on either *C. elegans* motility or pharyngeal activity (Table 1). These pathways regulate calcium signalling and membrane potential, two essential factors in *slo*-*1* function, which might provide the basis for an interaction of latrophilin and SLO-1 in emodepside’s effects. These studies are of interest as they may inform the development of combined therapies that incorporate additional drugs with emodepside to optimise the anthelmintic action in some target species of parasitic nematode. Indeed, some *C. elegans* mutations confer an increase in emodepside sensitivity, suggesting that antagonists of these pathways may merit further investigation as a means of providing synergy with the anthelmintic effects of emodepside (Willson et al. 2004; Amliwala 2005). The potential for modulation of SLO-1 core activity is consistent with modest but clear pharmacological regulation of emodepside-dependent activation of calcium-activated potassium channels in *A. suum* muscle (Buxton et al. 2011).

**Table 1:** A summary of genes that regulate emodepside sensitivity in the nematode *C. elegans*

Gene	Pharynx	Locomotion	Signalling cascade
*slo*-*1*	↓1000 fold	↓1000 fold	Ion channel
*lat1/lat2*	↓10-fold	No change	GPCR
*goa*-*1*	↑10-fold	↑50 fold	G-protein
*egl*-*30* (lof)	↓50-fold	↓3 fold	G-protein
*egl*-*30* (gof)	↑20-fold	↑20 fold	G-protein
*egl*-*8*	↓14-fold	↓10 fold	Phospholipase C
*unc*-*13*	↓40-fold	–	Transmitter release
*unc*-*10*	No change	–	Transmitter release
*snb*-*1*	↓30-fold	–	Transmitter release
*unc*-*31*	↓5-fold	–	Transmitter release

The sensitivity to emodepside of the *C. elegans* mutants listed was compared in parallel assays with the sensitivity of wild-type animals and the relative sensitivity of the mutant compared to wild-type expressed as a fold reduction (↓) or a fold increase (↑). ‘lof’ indicates a loss of function mutant and ‘gof’ indicates a gain-of-function mutant. The left-hand column indicates the mutants that were assayed. The effect of emodepside was determined in a pharyngeal assay in which extracellular electrophysiological recordings were made from the pharynx (Willson 2003; Willson et al. 2004; Bull 2007) and on locomotion in which the effect of emodepside on the frequency of locomotion on agar medium was determined (Amliwala 2005)

Whilst several pathways might impact on emodepside sensitivity, the fact that the *C. elegans* reverse genetic screen thus far only yielded *slo*-*1* mutants indicates that this is likely to be the major locus of action. This is also likely to be the case in parasitic nematodes as penitrem A, a selective calcium-activated potassium channel antagonist, potently blocks the paralytic effect of emodepside on *Ancylostoma caninum* (Welz et al. 2011). These observations indicate that SLO-1 is the major mediator of the anthelmintic action in parasites as well as *C. elegans* (Welz et al. 2011). Nonetheless, the relative role of *slo*-*1* and latrophilin in the efficacy of emodepside against different species of nematodes might be an avenue to explore further. In this regard, it is interesting to note that there is a precedent for a difference in anthelmintic action between species of nematode as ivermectin inhibits feeding in *A. suum* and *C. elegans* (Brownlee et al. 1997; Pemberton et al. 2001) through an action on glutamate-gated chloride channels; however, ivermectin does not affect the pharynx of *Ascaridia galli* (Holden-Dye and Walker 2006).

## Clues to the mode of action based on the time-course of action of emodepside

**Fig. 1:**
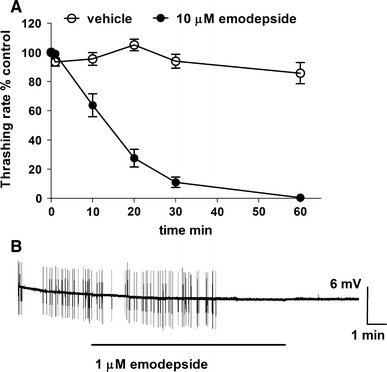
Examples of the time-course of the effect of emodepside on *C. elegans* motility and feeding. **a** The time-course of the inhibitory effect of emodepside on motility in liquid: control *n* = 10, emodepside *n* = 27, mean ± SE mean. The thrashing rate in each treatment group was measured at time 0 and then followed for a further 60 min at 10 min intervals. The thrashing rate at each time-point is expressed as a percentage of the initial thrashing rate at time 0. The vehicle was 0.1 % ethanol and the thrashing rate of this control group did not alter significantly over the time-course of the experiment (Amliwala 2005). **b** An example of the time-course of the effect of emodepside on pharyngeal activity in a cut head preparation. The recordings are extracellular electrophysiological recordings taken from the pharyngeal muscle. Each vertical deflection records a pharyngeal muscle contraction–relaxation cycle or pump. The *bar* indicates the duration of application of emodepside. Emodepside acts on SLO-1 in the pharyngeal circuit to inhibit pumping (Guest et al. 2007; Dillon et al. 2009). Note this experiment was performed in the absence of any 5-HT (which may be used to provide an excitation against which inhibition can be measured) and that in the absence of 5-HT the basal pumping rate in the cut head preparation is very slow. This recording illustrates the slow time to onset of the emodepside inhibition but the rapid manner in which pumping is turned off following this latency of onset. This can be explained by considering that at low rates of pumping, when the excitatory neuronal input to the pharynx is minimal, the inhibitory effect of emodepside is sufficient to turn pumping off completely. The data are taken from (Bull 2007)

Emodepside is a highly lipophilic compound with a logP value of 4.9 (octanol/water: http://sitem.herts.ac.uk/aeru/vsdb/Reports/1838.htm). With this in mind, it is interesting to note that the effect of emodepside on neuromuscular function, whether observed by behavioural analysis or by electrophysiological recordings, typically has a relatively long time-course for the development of the maximal effect. This has clearly been demonstrated for the effect on the body wall muscle of *A. suum* (Willson et al. 2003; Buxton et al. 2011). Also, for *C. elegans*, the indication is that emodepside has a slow time-course of action. For example, the motility of intact adult *C. elegans* immersed in saline containing emodepside is inhibited after 10 min but the maximum effect takes an hour to develop (Fig. 1a). The interpretation of this observation is confounded by the fact that in part this may be explained by the time taken for emodepside to gain access to the interior of the worm, either by ingestion or by crossing the cuticle. Nonetheless the slowly accumulating effect is consistent with the idea that once emodepside is distributed in the worm, it must then accumulate at the target site in order to elicit its effect. In a cut pharyngeal preparation from *C. elegans*, used to measure the effect on pharyngeal pumping, the cuticle no longer presents a barrier, and this can provide a clearer insight into the time-course of the effect. The inhibition of pharyngeal pumping by emodepside, either basal (Fig. 1b) (Crisford et al. 2011) or stimulated by 5-HT (Willson et al. 2004), takes minutes to appear whilst the effects of other bioactive hydrophilic agents, for example, either octopamine or the neuropeptide AF1 occurs rapidly, within seconds (Rogers et al. 2001). A plausible explanation for these observations on the time-course of the action of emodepside is that it reflects its hydrophobicity and as it partitions into the membrane, it gains access to a drug recognition site that lies in the transmembrane domains of the protein. Such a recognition site may well be within or between the helices that comprise the channel or at the protein/lipid interface within the bilayer. The nanomolar concentration in solution at which emodepside exerts behavioural inhibition of *C. elegans* suggests that it exerts its effects when the emodepside/lipid ratio (i.e. mole/mole ratio) is low. This supports the notion that a specific emodepside/protein or emodepside/protein/lipid interaction underpins its mode of action. A similar train of thought suggests that at micromolar concentrations of emodepside, a concentration that is difficult to keep in aqueous solution, emodepside would appear in the membrane at a 1:1 emodepside/lipid ratio where one might expect to see additional membrane perturbant effects. Indeed, this expectation is realised by the report that the parent compound of emodepside, PF1022A, has ionophore properties and directly confers ion channel activity on membranes, although this appears unrelated to its anthelmintic action (Geßner et al. 1996; Dornetshuber et al. 2009).

Although the biophysical nature of emodepside strongly supports a membrane-associated binding site, this does not a priori predict a slow time-course on the biological action. It is informative to consider that local anaesthetics that have to partition into the membrane to elicit their biological effect may exhibit a slow time-course that is dictated by their rate of entry into the membrane, and this may also be the situation for emodepside. Alternatively the rate of onset of the biological effect of emodepside could reflect its rate of access and binding to its target site within the membrane. An additional possibility that would influence the time-course of the biological effect is that emodepside may preferentially bind to a specific conformation of the SLO-1 channel, that is, it may exhibit state-dependent binding to its target. There are a number of precedents for this latter interaction in which drugs bind within the membrane to open or allosterically modified ion channel or transporter conformations, for example, ouabain (Qiu et al. 2005; Sandtner et al. 2011) and thapsigargin (Winther et al. 2010). Whatever the precise interaction of emodepside with the calcium-activated potassium channel, it is clear from the various experimental observations to date that its biological effect is exerted over a prolonged time-course and understanding the mechanistic underpinning of this phenomenon will provide further insight into its mode of action.

## Does emodepside interact directly with SLO-1?

**Fig. 2:**
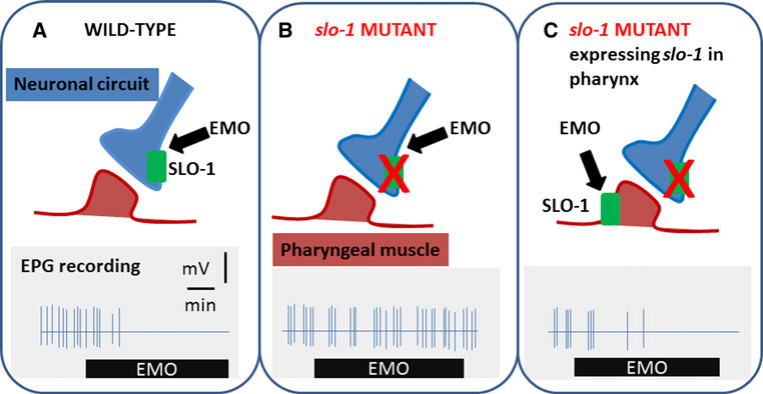
Activation of SLO-1 by emodepside. A cartoon to illustrate data from Crisford et al. (2011) which showed that ectopic expression of *slo*-*1* in *C. elegans* pharynx conferred sensitivity to emodepside in an otherwise emodepside resistant strain*, slo*-*1 (js379)*, consistent with the proposal that emodepside interacts with the channel. **a** Wild-type *C. elegans* express *slo*-*1* in pharyngeal neurones and not muscle (Chiang et al. 2006). Application of emodepside inhibits pharyngeal activity (recorded by an electropharyngeogram, EPG) by interfering with signalling in the pharyngeal circuit in a *slo*-*1* dependent manner (Guest et al. 2007). **b** The *slo*-*1* null mutant has an altered pattern of pharyngeal activity that consists of bursts of pumps occurring together, represented by the cartoon of the EPG. This reflects the role of *slo*-*1* in patterning the activity in the neural circuit that drives feeding behaviour (Dillon et al. 2009). However, the *slo*-*1* mutant is completely resistant to the inhibitory effects of emodepside, consistent with its pivotal in the mode of drug action (Dillon et al. 2009; Crisford et al. 2011). **c** Ectopic expression of *slo*-*1* from the pharyngeal muscle-specific promoter *myo*-*2* fails to rescue the disrupted pattern of pharyngeal activity (i.e. the bursting activity is still observed) in line with earlier observations that this is a neuronally derived phenotype; however, it does confer sensitivity of the pharynx to emodepside. This is consistent with the suggestion that emodepside can signal through SLO-1 when it is ectopically expressed in the muscle to inhibit activity

The profound drug resistance imparted by the *slo*-*1* loss of function mutations in *C. elegans* (Guest et al. 2007) strongly suggests that SLO-1 harbours the emodepside binding site. Furthermore, genetic analysis and physiological observations are consistent with the idea that emodepside acts to open the channel. This proposal also marries well with electrophysiological recordings of the effect of emodepside on *A. suum* muscle (Willson et al. 2003; Buxton et al. 2011) which are consistent with opening of a calcium-activated potassium conductance. There are at least two alternative mechanisms through which emodepside could exert this action. It could influence the dependence of the SLO-1 channel on membrane potential (depolarisation) and/or its regulation by intracellular calcium ions (Salkoff et al. 2006). Furthermore, the molecular organisation of SLO-1 channels and their mammalian orthologues suggest that in addition to the core tetramer of SLO-1 α subunits that form the ion channel, there are a number of associated β subunits and scaffolding proteins that support function (Salkoff et al. 2006) through which emodepside could also exert its effect. Recently such an accessory subunit, designated BKIP, has been identified in *C. elegans* (Chen et al. 2010). Thus, nematode SLO-1 channels may co-assemble with accessory subunits in a similar fashion to the mammalian channels, and this may impart drug sensitivity. Nonetheless, experiments in which recombinant *slo*-*1* is heterologously expressed demonstrate that the core SLO-1 α-subunit tetramer can reconstitute a calcium- and voltage-activated channel that is emodepside sensitive. Preliminary data from this work, reported at the Ion Channel Symposium, Philadelphia 2011 (Crisford, Holden-Dye, O’Kelly, Harder, Welz, O’Connor and Walker), showed that emodepside facilitates activation of SLO-1 channels heterologously expressed in HEK293 cells. This is consistent with an earlier study in which the sensitivity of *C. elegans* pharynx to emodepside in a *slo*-*1* null mutant was conferred by ectopic over-expression of *slo*-*1* (Crisford et al. 2011) (Fig. 2). Taken together these data suggest that SLO-1 can impart a molecular target with sensitivity to emodepside. That this is achieved via expression of the α subunit suggests that this moiety involving the tetrameric assembly of the SLO-1 channel harbours the likely target site, which will be in the membrane or at an interface between the membrane and the extracellular or intracellular compartment.

**Fig. 3:**
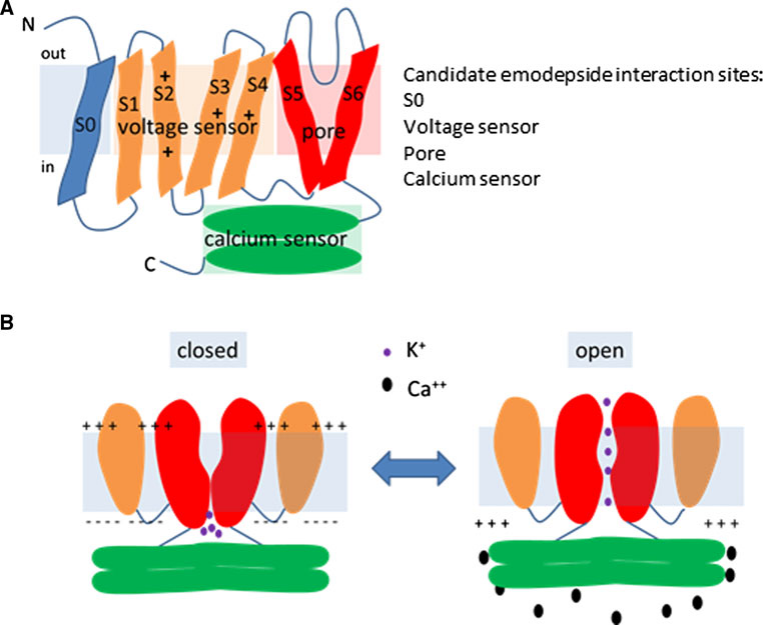
A schematic of the structure of the SLO-1/BK calcium-activated potassium channel. This is based on the information from papers that have reported the structural properties of the human BK channel (Latorre et al. 2010; Pantazis et al. 2010; Yuan et al. 2010, 2012) and a review of these data (Latorre et al. 2010). **a** A single α subunit of the SLO-1 channel showing the relationship between the voltage sensor domain, pore forming domain and calcium sensing domain. S0 is the least conserved region between the different isoforms of the channel, whereas the pore forming and calcium sensing domains are highly conserved. The transmembrane segments S2, S3 and S4 harbour charged residues as indicated which confer the voltage sensitivity of the channel. **b** A simplified diagram (omitting S0) showing the relationship between two α subunits of the SLO-1 tetramer and the opening of the channel

As the likely action of emodepside is to facilitate channel function, one might predict that it interacts allosterically to modulate the channel by binding to voltage or calcium sensing domains or the gating mechanisms that control SLO-1 channel opening. The location of the functional domains in the BK channel has recently been reviewed (Latorre et al. 2010) (Fig. 3). Interestingly, regions within these domains, especially the calcium sensor or ‘calcium bowl’ (Crisford et al. 2011), are among the most conserved among the mammalian and nematode SLO-1 channels. The binding sites for a number of toxins that interact with BK channels have been mapped onto this overall structure. The interactions of charybdotoxin, iberiotoxin and tetraethylammonium (TEA) are determined by the extracellular face of the pore forming domains of the channel, S5 and S6 (MacKinnon and Miller 1989; Mullmann et al. 1999). However, the hydrophobicity of emodepside and its prolonged time-course of action suggest that rather than act at a similar extracellular facing site as these aforementioned toxins, it may access a binding site on the channel within the lipid membrane. The membrane harbours the domain that confers voltage sensing capability on the ion channel protein. This comprises four positively charged residues, two in the second transmembrane domain (S2), one in the third transmembrane domain (S3) and one in the fourth transmembrane domain (S4) (Ma et al. 2006). Recently, it has been shown that there is a cooperative interaction between S2 and S4 in terms of conferring voltage sensitivity on the channel (Pantazis et al. 2010). Thus, an interesting possibility is that emodepside may exert its action within the membrane at the lipid–protein interface between S2 and S4 to modulate voltage sensing. There is a clear precedent for this kind of interaction provided by an analysis of the binding of a toxin from Chilean Rode Tarantula to the potassium channel KvAP. This toxin accesses the voltage sensor domain that lies within the membrane (Lee and MacKinnon 2004). Indeed, there is a growing appreciation that drug binding sites for ion channels may reside within the hydrophobic environment at the lipid–protein interface.

The cytoplasmic domain of the BK channel provides the calcium sensor which consists of a gating ring of two domains per subunit, that is, eight in total in the functional tetrameric channel, termed RCK for regulator of potassium conductance. Structural studies have provided information on the conformation of the closed gating ring (Wu et al. 2010) and the calcium-bound activated conformation (Yuan et al. 2012). Intriguingly, a cluster of mutations that confer resistance to emodepside were mapped to the RCK domains, comprising the gating ring, of SLO-1 (Guest et al. 2007). This suggests significant determinants of efficacy (and possible binding sites for emodepside) exist at the protein–lipid interface of this domain. A speculative note is that emodepside might interpose between the intracellular gating ring and the pore domain to modulate channel activity. Where ever the site of interaction of emodepside within the SLO-1 channel might lie, defining it will have important potential for understanding the molecular basis of the mode of action of emodepside.

## Summary

The means of targeting parasitic nematodes relies on a relatively restricted pharmacological armoury of anthelmintics: The cyclo-octadepsipeptide emodepside provides a completely new pharmacological strategy that breaks resistance acquired to other anthelmintic agents. SLO-1 is the most significant player in its mode of action. Moreover, it belongs to a family of channels that are highly conserved through the animal phyla and implicated in a range of therapeutic strategies and neurological disorders. Thus, not only does understanding the mode of action of emodepside provide a new anthelmintic strategy, it also has the potential to provide a novel route for other pharmacotherapies that require the capability to modulate neuronal or muscle excitability (N’Gouemo 2011).

## References

[CR1] Amliwala K (2005) Molecular and genetic determinants of the inhibitory action of emodepside on *C. elegans muscle*. Division of Cell Sciences 2005, University of Southampton PhD Thesis: Southampton. p 148

[CR2] Brownlee DJ, Holden-Dye L, Walker RJ (1997). Actions of the anthelmintic ivermectin on the pharyngeal muscle of the parasitic nematode, *Ascaris suum*. Parasitology.

[CR3] Bull K (2007) An investigation into the action of emodepside and other novel anthelmintic compounds. Division of Cell Sciences 2007, University of Southampton PhD Thesis: Southampton. p 275

[CR4] Bull K, Cook A, Hopper NA, Harder A, Holden-Dye L, Walker RJ (2007). Effects of the novel anthelmintic emodepside on the locomotion, egg-laying behaviour and development of *Caenorhabditis elegans*. Int J Parasitol.

[CR5] Buxton SK, Neveu C, Charvet CL, Robertson AP, Martin RJ (2011). On the mode of action of emodepside: Slow effects on membrane potential and voltage-activated currents in *Ascaris suum*. Brit J Pharmacol.

[CR6] Chen B, Ge Q, Xia X-M, Liu P, Wang SJ, Zhan H, Eipper BA, Wang Z-W (2010). A novel auxiliary subunit critical to BK channel function in *Caenorhabditis elegans*. J Neurosci.

[CR7] Chiang J, Steciuk M, Shtonda B, Avery L (2006). Evolution of pharyngeal behaviours and neuronal functions in free-living soil nematodes. J Exp Biol.

[CR8] Crisford A, Murray C, O’Connor V, Edwards RJ, Kruger N, Welz C, von Samson-Himmelstjerna G, Harder A, Walker RJ, Holden-Dye L (2011). Selective toxicity of the anthelmintic emodepside revealed by heterologous expression of human KCNMA1 in *Caenorhabditis elegans*. Mol Pharmacol.

[CR9] Dillon J, Andrianakis I, Bull K, Glautier S, O’Connor V, Holden-Dye L, James C (2009). AutoEPG: software for the analysis of electrical activity in the microcircuit underpinning feeding behaviour of *Caenorhabditis elegans*. PLoS ONE.

[CR10] Dornetshuber R, Kamyar MR, Rawnduzi P, Baburin I, Kouri K, Pilz E, Hornbogen T, Zocher R, Berger W, Lemmens-Gruber R (2009). Effects of the anthelmintic drug PF1022A on mammalian tissue and cells. Biochem Pharmacol.

[CR11] Geßner G, Meder S, Rink T, Boheim G, Harder A, Jeschke P, Scherkenbeck J, Londershausen M (1996). Ionophore and anthelmintic activity of PF 1022A, a cyclooctadepsipeptide, are not related. Pesticide Sci.

[CR12] Glauser DA, Johnson BE, Aldrich RW, Goodman MB (2011). Intragenic alternative splicing coordination is essential for *Caenorhabditis elegans**slo*-*1* gene function. PNAS.

[CR13] Guest M, Bull K, Walker RJ, Amliwala K, O’Connor V, Harder A, Holden-Dye L, Hopper NA (2007). The calcium-activated potassium channel, SLO-1, is required for the action of the novel cyclo-octadepsipeptide anthelmintic, emodepside, in *Caenorhabditis elegans*. Int J Parasitol.

[CR14] Holden-Dye L, Walker RJ (2006). Actions of glutamate and ivermectin on the pharyngeal muscle of *Ascaridia galli:* A comparative study with *Caenorhabditis elegans*. Int J Parasitol.

[CR15] Holden-Dye L, O’Connor V, Hopper N, Walker R, Harder A, Bull K, Guest M (2007). SLO, SLO, quick, quick, slow: calcium-activated potassium channels as regulators of *Caenorhabditis elegans* behaviour and targets for anthelmintics. Invert Neurosci.

[CR16] Johnson BE, Glauser DA, Dan-Glauser ES, Halling DB, Aldrich RW, Goodman MB (2011). Alternatively spliced domains interact to regulate BK potassium channel gating. PNAS.

[CR17] Krucken J, Harder A, Jeschke P, Holden-Dye L, von Samson-Himmelstjerna G (2012) Emodepside- an anthelmintic with a new mode of action*.* Trends Parasitol (submitted) 10.1016/j.pt.2012.06.005 22858281

[CR18] Krüger N, Harder A, von Samson-Himmelstjerna G (2009). The putative cyclooctadepsipeptide receptor depsiphilin of the canine hookworm *Ancylostoma caninum*. Parasitol Res.

[CR19] Latorre R, Morera FJ, Zaelzer C (2010). Allosteric interactions and the modular nature of the voltage- and Ca^2+^-activated (BK) channel. J Physiol.

[CR20] Lee SY, MacKinnon R (2004). A membrane-access mechanism of ion channel inhibition by voltage sensor toxins from spider venom. Nature.

[CR21] Lelianova VG, Davletov BA, Sterling A, Rahman MA, Grishin EV, Totty NF, Ushkaryov YA (1997). α-Latrotoxin receptor, latrophilin, is a novel member of the secretin family of G protein-coupled receptors. J Biol Chem.

[CR22] Ma Z, Lou XJ, Horrigan FT (2006). Role of charged residues in the S1–S4 voltage sensor of BK channels. J Gen Physiol.

[CR23] MacKinnon R, Miller C (1989). Mutant potassium channels with altered binding of charybdotoxin, a pore-blocking peptide inhibitor. Science.

[CR24] Mullmann TJ, Munujos P, Garcia ML, Giangiacomo KM (1999). Electrostatic Mutations in iberiotoxin as a unique tool for probing the electrostatic structure of the maxi-K channel outer vestibule. Biochemistry.

[CR25] N’Gouemo P (2011). Targeting BK (big potassium) channels in epilepsy. Exp Opinion Therap Targets.

[CR26] Pantazis A, Gudzenko V, Savalli N, Sigg D, Olcese R (2010). Operation of the voltage sensor of a human voltage- and Ca2+-activated K+ channel. PNAS.

[CR27] Pemberton D, Franks C, Walker R, Holden-Dye L (2001). Characterization of glutamate-gated chloride channels in the pharynx of wild-type and mutant *Caenorhabditis elegans* delineates the role of the subunit GluCl-alpha2 in the function of the native receptor. Mol Pharmacol.

[CR28] Qiu LY, Krieger E, Schaftenaar G, Swarts HGP, Willems PHGM, De Pont JJHHM, Koenderink JB (2005). Reconstruction of the complete ouabain-binding pocket of Na,K-ATPase in gastric H,K-ATPase by substitution of only seven amino acids. J Biol Chem.

[CR29] Rogers C, Franks C, Walker R, Burke J, Holden-Dye L (2001). Regulation of the pharynx of *Caenorhabditis elegans* by 5-HT, octopamine, and FMRFamide-like neuropeptides. J Neurobiol.

[CR30] Saeger B, Schmitt-Wrede H-P, Dehnhardt M, Benten WPM, Krücken J, Harder A, von Samson-Himmelstjerna G, Wiegand H, Wunderlich F (2001). Latrophilin-like receptor from the parasitic nematode *Haemonchus contortus* as target for the anthelmintic depsipeptide PF1022A. FASEB J.

[CR31] Salkoff L, Butler A, Ferreira G, Santi C, Wei A (2006). High-conductance potassium channels of the SLO family. Nat Rev Neurosci.

[CR32] Sandtner W, Egwolf B, Khalili-Araghi F, Sánchez-Rodríguez JE, Roux B, Bezanilla F, Holmgren M (2011). Ouabain binding site in a functioning Na+/K+ ATPase. J Biol Chem.

[CR33] Silva J-P, Lelianova VG, Ermolyuk YS, Vysokov N, Hitchen PG, Berninghausen O, Rahman MA, Zangrandi A, Fidalgo S, Tonevitsky AG, Dell A, Volynski KE, Ushkaryov YA (2011). Latrophilin 1 and its endogenous ligand Lasso/teneurin-2 form a high-affinity transsynaptic receptor pair with signaling capabilities. PNAS.

[CR34] von Samson-Himmelstjerna G, Harder A, Sangster NC, Coles GC (2005). Efficacy of two cyclooctadepsipeptides, PF1022A and emodepside, against anthelmintic-resistant nematodes in sheep and cattle. Parasitology.

[CR35] Welz C, Krüger N, Schniederjans M, Miltsch SM, Krücken J, Guest M, Holden-Dye LM, Harder A, von Samson-Himmelstjerna G (2011). SLO-1-channels of parasitic nematodes reconstitute locomotor behaviour and emodepside sensitivity in *Caenorhabditis elegans slo*-*1* loss of function mutants. PLoS Pathog.

[CR36] Willson JM (2003) An investigation into the mode of action of the novel anthelmintic emodepside. Cell sciences division PhD Thesis, University of Southampton: Southampton. p 201

[CR37] Willson J, Amliwala K, Harder A, Holden-Dye L, Walker RJ (2003). The effect of the anthelmintic emodepside at the neuromuscular junction of the parasitic nematode *Ascaris suum*. Parasitology.

[CR38] Willson J, Amliwala K, Davis A, Cook A, Cuttle MF, Kriek N, Hopper NA, O’Connor V, Harder A, Walker RJ, Holden-Dye L (2004). Latrotoxin receptor signaling engages the UNC-13-dependent vesicle-priming pathway in *C. elegans*. Curr Biol.

[CR39] Winther A-ML, Liu H, Sonntag Y, Olesen C, le Maire M, Soehoel H, Olsen C-E, Christensen SB, Nissen P, Møller JV (2010). Critical roles of hydrophobicity and orientation of side chains for inactivation of sarcoplasmic reticulum Ca^2+^-ATPase with thapsigargin and thapsigargin analogs. J Biol Chem.

[CR40] Wu Y, Yang Y, Ye S, Jiang Y (2010). Structure of the gating ring from the human large-conductance Ca^2+^-gated K^+^ channel. Nature.

[CR41] Yuan P, Leonetti MD, Pico AR, Hsiung Y, MacKinnon R (2010). Structure of the human BK channel Ca^2+^-activation apparatus at 3.0 Å resolution. Science.

[CR42] Yuan P, Leonetti MD, Hsiung Y, MacKinnon R (2012). Open structure of the Ca^2+^ gating ring in the high-conductance Ca^2+^-activated K^+^ channel. Nature.

